# Rapid Determination of Positive–Negative Bacterial Infection Based on Micro-Hyperspectral Technology

**DOI:** 10.3390/s24020507

**Published:** 2024-01-13

**Authors:** Jian Du, Chenglong Tao, Meijie Qi, Bingliang Hu, Zhoufeng Zhang

**Affiliations:** 1 Key Laboratory of Spectral Imaging Technology CAS, Xi’an Institute of Optics and Precision Mechanics, Chinese Academy of Sciences, Xi’an 710119, China; dujian@opt.ac.cn (J.D.);; 2 Xi’an Key Laboratory for Biomedical Spectroscopy, Xi’an 710119, China

**Keywords:** micro-hyperspectral technology, bacterial infection, positive–negative determination, spectral feature, directly smeared urine sample, deep learning

## Abstract

To meet the demand for rapid bacterial detection in clinical practice, this study proposed a joint determination model based on spectral database matching combined with a deep learning model for the determination of positive–negative bacterial infection in directly smeared urine samples. Based on a dataset of 8124 urine samples, a standard hyperspectral database of common bacteria and impurities was established. This database, combined with an automated single-target extraction, was used to perform spectral matching for single bacterial targets in directly smeared data. To address the multi-scale features and the need for the rapid analysis of directly smeared data, a multi-scale buffered convolutional neural network, MBNet, was introduced, which included three convolutional combination units and four buffer units to extract the spectral features of directly smeared data from different dimensions. The focus was on studying the differences in spectral features between positive and negative bacterial infection, as well as the temporal correlation between positive–negative determination and short-term cultivation. The experimental results demonstrate that the joint determination model achieved an accuracy of 97.29%, a Positive Predictive Value (PPV) of 97.17%, and a Negative Predictive Value (NPV) of 97.60% in the directly smeared urine dataset. This result outperformed the single MBNet model, indicating the effectiveness of the multi-scale buffered architecture for global and large-scale features of directly smeared data, as well as the high sensitivity of spectral database matching for single bacterial targets. The rapid determination solution of the whole process, which combines directly smeared sample preparation, joint determination model, and software analysis integration, can provide a preliminary report of bacterial infection within 10 min, and it is expected to become a powerful supplement to the existing technologies of rapid bacterial detection.

## 1. Introduction

In recent years, the rampant spread of pathogenic microorganisms has posed significant challenges to public health [1]. According to the data released by the World Health Organization (WHO), deaths from infectious diseases account for 19% of the total global mortality, with approximately 13 million children succumbing to infectious diseases each year [2,3]. The experiences of large medical institutions in dealing with infectious diseases and major epidemics have told us that early detection and diagnosis are the keys to effectively treating infections and controlling epidemics. It is crucial to detect pathogenic bacteria more rapidly and accurately in the early stage [4,5].

However, the current bacterial detection techniques have disadvantages such as long bacterial culture periods and inadequate detection throughput, making it difficult to meet the clinical demand for rapid detection [6], especially for sterile body fluids with low bacterial content (such as blood, pleural fluid, cerebrospinal fluid, etc.). These samples require processes like bacterial enrichment through blood culture, positive-culturing onto blood plates, and isolation identification, which collectively results in low overall testing efficiency [7,8]. For the most time-consuming bacterial culture process (usually taking 1–2 days), a positive result is usually reported when the bacterial suspension concentration exceeds 10^5^ CFU/mL (Colony Forming Units per milliliter) after blood bottle culture, while cases with concentrations less than 10^3^ CFU/mL are usually presumed negative and do not require further testing. In clinical testing, we have observed that during these processes, specifically before or in the early stages of blood culture, if the infection status (positive or negative) directly from the samples can be accurately determined, it can significantly reduce the overall testing time. This means that positive samples can enter the antimicrobial susceptibility testing phase as soon as possible. For negative samples, there is no need for subsequent continuous cultivation to save consumables like culture plates and other medical supplies. In particular, there is a huge demand for urine sample testing in the emergency department, where the rapid issuance of bacterial test reports is of great significance [9,10]. In other words, for the test sample, we aim to directly detect whether it contains bacteria. If bacteria are detected, it is positive, and if there are no bacteria, it is negative. This process requires eliminating interference from impurities and other substances in the urine that might affect the bacterial target.

Therefore, we aim to introduce new technology in the determination of bacterial infection status of directly smeared urine samples to accelerate the detection process and reduce costs. Hyperspectral imaging (HSI) has developed from multispectral imaging, using imaging spectrometers to continuously image target objects in dozens or hundreds of spectral bands from ultraviolet to near-infrared (200–2500 nm) [11,12]. HSI has been widely applied in the field of remote sensing, such as terrain classification [13], agricultural monitoring [14], and food safety [15]. The micro-hyperspectral imaging technology that has emerged in recent years is a combination of spectral analysis technology and microscopic imaging technology. Through the meticulous segmentation of spectral bands, higher-resolution, continuous, and narrow-band micro-hyperspectral images can be obtained, enabling a comprehensive analysis of qualitative, quantitative, and localization of microscopic tissue.

In the field of medical spectral research, the current spectral resolution of micro-hyperspectral imaging systems can reach 3 nm, with spatial resolution exceeding 0.5 μm [16]. With the continuous improvement of various hardware parameters, it is possible to monitor pathophysiological characteristics and classify bacterial genera and species [17]. Bacteria are mainly composed of proteins, nucleic acids, lipids, carbohydrates, and coenzymes, and different components have their own typical wavelength selectivity. The specific absorbers have strong absorption characteristics for certain specific wavelengths. The variations in the content of these substances can result in differential degrees of absorption, reflection, and scattering of light waves, ultimately manifesting as distinctive spectral features between bacterial genera, which provides a theoretical foundation for hyperspectral research of bacteria [18,19].

At present, hyperspectral research on bacteria mostly focuses on the classification and identification of several specific types of bacteria. For example, Matthew employed micro-hyperspectral imaging to detect Salmonella in chicken rinsate [20]. The hyperspectral data of the Salmonella colony at 100× magnification within the wavelength range of 450–800 nm was obtained. A classification accuracy of 98.5% and specificity of 0.963 was achieved by using Quadratic Discriminant Analysis (QDA). Through a combination of micro-hyperspectral imaging and machine learning, Liu achieved a classification accuracy of 98.06% for two types of bacilli, *B. megaterium* and *B. cereus*, based on subtle differences in absorption peaks [21]. Kang utilized frameworks such as Convolutional Neural Network (CNN) and Long Short-Term Memory (LSTM) and made significant progress in the classification of foodborne bacteria [22,23]. The Fusion-net proposed stacked single-analysis frameworks and completed the synchronous processing of multiple features, improving the classification accuracy to 98.4%. However, most of his research is confined to a few specific types of foodborne bacteria. The narrow wavelength and low spectral resolution lack enough biological information for more complex multi-class detection of infectious bacteria [24,25]. Moreover, small-scale datasets make it difficult to accurately reflect the actual clinical distribution of bacteria [26]. Tao designed an end-to-end deep learning network by combining micro-hyperspectral imaging systems to extract species-specific features at the bacterial level as bacterial differentiation fingerprints [27]. A classification model for common bacteria was established based on a large-scale dataset, and the accuracy of classification for uncommon bacteria was achieved at 92% via transfer learning.

The above research indicates that bacterial detection technology based on micro-hyperspectral imaging has the capability to encode the biological characteristics of bacteria into datacubes via spectral and morphological information representation at the microscopic scale. By employing appropriate preprocessing methods and deep learning models, more detailed and intricate deep spectral features can be extracted. Compared with existing bacterial detection methods, micro-hyperspectral technology offers simplicity in operation and saves a significant amount of cultivation time. Moreover, it does not rely on traditional morphological observation, which can reduce the influence of human factors. Compared with similar studies on bacterial hyperspectral analysis, this study has made progress in the research object, research methods, and application of results. The research scope extends to a broader range of clinical infectious bacteria. The multi-scale buffered convolutional neural network has powerful capabilities of multi-dimensional feature extraction. This study has better scalability and higher application efficiency. Micro-hyperspectral technology is expected to become a reliable means to address the issue of rapid bacterial detection. However, as for the rapid determination of directly smeared bacterial infection status proposed in this study, there are currently no relevant research outcomes that have been observed.

Therefore, this study took a unique perspective on the bacterial infection status of directly smeared samples. Focusing on the common urine samples in clinical practice, this study discussed the features of hyperspectral bacteria data in directly smeared conditions, the differences in spectral features between positive and negative bacterial infection, deep learning models suitable for multi-scale features and rapid analysis of directly smeared data, as well as the temporal correlation between positive–negative determination and short-term cultivation. This study established a standard spectral database for common bacteria (*Escherichia*, *Enterococcus*, *Staphylococcus*, *Candida*, etc.) and impurities (crystal, casts, etc.) in urine samples to eliminate interference from impurities, and realized spectral matching with single-bacterium targets. Based on the hyperspectral data characteristics of directly smeared samples, a multi-scale buffered convolutional neural network, the Multi-BufferNet (abbreviated as MBNet), was established, which included three convolutional combination units to extract the spectral features of directly smeared data from different dimensions. Finally, a model was established by combining database matching and MBNet, called the joint determination model, which achieved rapid and accurate prediction of urine bacterial infection. To apply this technology to clinical outpatient practice, this study also combined the front-end rapid preparation method of directly smeared urine samples and the back-end automated analysis reporting software, exploring a more efficient and feasible determination solution for the whole process. This study, in conjunction with the genus identification step [27], has formed a complete and rapid bacterial determination process.

## 2. Materials and Methods

### 2.1. Micro-Hyperspectral Imaging System

**Figure 1:**
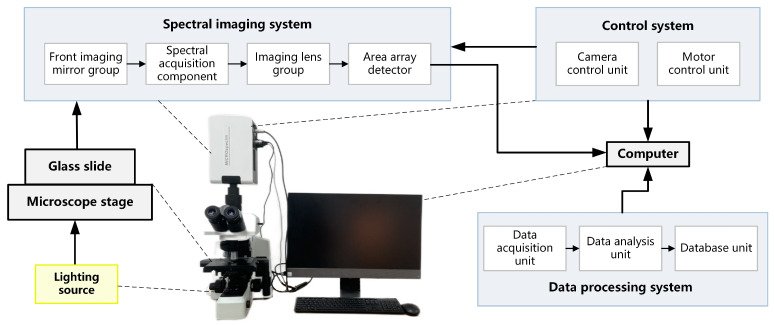
Structural schematic diagram of a micro-hyperspectral imaging system.

The data used in this study were all acquired by the micro-hyperspectral imaging system, MICROspecim. MICROspecim consists of a spectral imaging system, control system, and data processing system, as shown in Figure 1. The spectral imaging system includes a front imaging mirror group, spectral acquisition component, imaging lens group, and area array detector. The control system includes a camera control unit and a motor control unit. The data processing system includes a data acquisition unit, a data analysis unit, and a database unit. A halogen lamp (400–2500 nm, 50 W) provides an active lighting source. The glass slide samples on the microscope stage are imaged on the area array detector through the spectral imaging system to complete two-dimensional information acquisition. Simultaneously, the control system operates the motor to complete another one-dimensional spatial information scanning. The control system and data processing system are uniformly integrated into computer software, responsible for datacube acquisition and post-processing. As a result, the hyperspectral data of directly smeared bacterial sample is obtained with a dimension of 226 (*λ*) × 800 (*x*) × 800 (*y*), where 800 × 800, which represents the image size (physical area size of 0.12 mm × 0.12 mm), and 226 is the number of spectral channels from 400 nm to 1000 nm. At present, MICROspecim has been applied in clinical pathology-assisted diagnosis and rapid bacterial analysis research.

### 2.2. Experimental Samples Preparation

**Figure 2:**
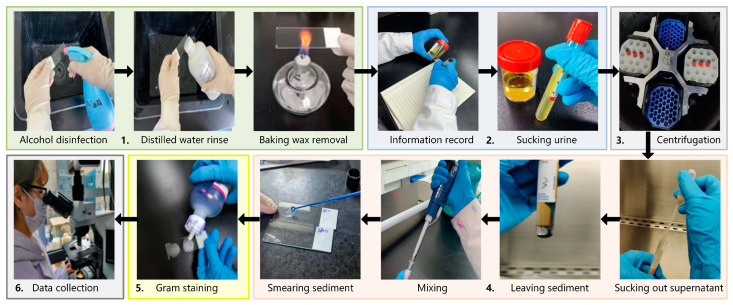
The preparation process of directly smeared urine samples.

In this study, urine-smeared slides were used as experimental samples. After obtaining patients’ urine samples, a portion was taken as the experimental group for directly smeared urine sample preparation. Another portion served as the control group, and the urine sample was determined and labeled as either a positive or negative bacterial infection sample using the traditional culture test process. Moreover, the positive samples also needed to be labeled with information about the bacterial species they contained. This information served as the ground truth for training samples. The preparation process of directly smeared urine samples in the experimental group is shown in Figure 2:

1. Take a clean glass slide, disinfect it with alcohol, and rinse it with distilled water. Then, bake it with an alcohol lamp to remove wax and cool it for later use.
2. Record detailed information on the urine sample and assign it a unique identifier. Pour the urine into an anticoagulant tube and balance it (so that the fluid volume in each tube is approximately the same).
3. Place the urine sample in a centrifuge and spin it at a speed of 3000 r/10 min.
4. Take out the centrifuged urine and use a clean sterile pipette to suck out the supernatant, leaving urine sediment at the bottom. Then, use a new pipette to suck out the urine sediment and mix it thoroughly. Smear the urine sediment on a slide and spread it quickly and evenly by a sterile loop.
5. Place the prepared slide in a biosafety cabinet until it is completely dry. Then, proceed with Gram-staining in the following order: stain with crystal violet, cover with iodine, decolorize with 95% ethanol, and counterstain with safranine. Finally, rinse the slide with water and air-dry it for later use. The Gram-staining process is necessary for two reasons. First, Gram-staining is an inherent part of the current testing process, which can highlight the morphological information of bacterial targets and facilitate doctors during observation and determination. It is beneficial for our technology to adhere to the existing bacterial testing process to the maximum extent possible. Second, the bacterial profile and detailed information of the unstained sample are not clear enough without Gram-staining. It is challenging for doctors to label specific bacteria or impurities.
6. Place the slide on the microscope stage and search for the field of view under a 10× objective. Convert the objective lens to a 100× objective lens and look for a field of view suspected to contain bacterial distribution. Then, perform a push scanning to capture hyperspectral images of directly smeared urine samples.

For the urine samples of the control group, after traditional culture, staining, biochemical molecular diagnosis, and mass spectrometry, the true value of bacterial infection status is determined. The experimental group is labeled with the corresponding sample identifiers.

### 2.3. Experimental Dataset

**Figure 3:**
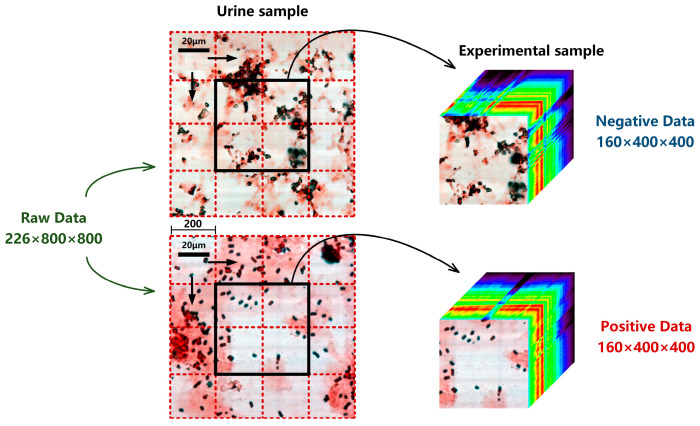
Extraction process of experimental sample from a raw urine sample. Each small red dashed box represents a spatial stride of 200 × 200, and each displacement can obtain an experimental sample. The black boxes represent the spatial size (400 × 400) of the experimental sample.

The urine samples in this study were all from the Clinical Laboratory of Tangdu Hospital. The experimental dataset was collected by MICROspecim, including 8124 sets of urine sample data, as shown in Table 1. Among them, 2864 cases are negative (sterile) samples of bacterial infections and 5260 are positive samples. The largest sample size among positive samples is *E. coli* (1442 cases), followed by *E. faecalis* (720), *C. tropicalis* (594), *K. pneumoniae* (510), *C. albicans* (460), P. mirabilis (365), S. epidermidis (322), *P. aeruginosa* (315), *S. aureus* (296), and *A. baumannii* (236). Each set of urine samples includes 0 h (no cultivation, abbreviated as 0 h) and 3 h (short-term cultivation, abbreviated as 3 h) samples. The data size of each raw urine sample is 226 × 800 × 800. The first 26 and last 40 spectral bands of data need to be removed due to the high noise level, retaining only the visible and near-infrared spectral data from 450–900 nm. The spatial dimension size (800 × 800) of raw sample data is relatively large, which is not conducive to model construction and training. Therefore, in this study, a spatial stride of 200 was used to extract experimental data (160 × 400 × 400) from the raw data as an experimental sample set, as shown in Figure 3. After three horizontal and vertical displacements, the total amount of experimental data was expanded to 9 × 8124 = 73,116. Finally, each sample was reviewed, problematic data were removed, and the truth values (positive or negative) of ultimate infection status were determined.

### 2.4. Database Standardization

The raw hyperspectral data of directly smeared bacteria is susceptible to factors such as system light source, optical components, and experimental environment, resulting in some random or systematic errors in the spectral and spatial dimensions. Therefore, when obtaining raw sample data, it is necessary to perform database standardization preprocessing to eliminate the impact of the system and external environment [28]. The main steps include the following:

1. Maintain the light source intensity, focal length, and magnification constant, and collect hyperspectral image *B*_1_ of the blank sample from a blank area on the slide.
2. Calculate the correction coefficient of spectral dimension:

(1) $$B(i,j)=\frac{\sum_{\lambda =1}^{N}B_{1}(i,j,\lambda )}{N}$$

(2) $$h_{1}(i,j,\lambda )=\frac{B(i,j)}{B_{1}(i,j,\lambda )}$$

(i,j,λ) represents the coordinates of a pixel on the spectral image of a blank sample; i,j, and λ represent the coordinates of its spatial two dimension and spectral dimension, respectively. *N* is the number of the spectral bands, and *B* is the average hyperspectral data of the blank sample in all spectral bands. $h_{1}(i,j,\lambda )$ is the correction coefficient of spectral dimension corresponding to the pixel at (i,j,λ).

3. Calculate the correction coefficient of spatial dimension:

(3) $$h_{2}(i,j,\lambda )=\frac{\sum_{i=1}^{P}\sum_{j=1}^{Q}B_{1}(i,j,\lambda )}{P\times Q}$$

*P* and *Q* are the number of horizontal and vertical pixels in the spectral image of a blank sample, respectively. $h_{2}(i,j,\lambda )$ is the correction coefficient of spatial dimension corresponding to the pixel at (i,j,λ).

4. Joint spatial and spectral dimension correction to obtain standardized hyperspectral data:

(4) $$S^{\prime }=\frac{h_{1}\times h_{2}\times S}{B_{1}}$$

S is the raw hyperspectral data, and $S^{\prime }$ is the standardized hyperspectral data obtained after S is corrected.

### 2.5. Spectral Angle Matching

Spectral Angle Matching (SAM) is a method used for spectral data analysis and comparison, commonly employed in tasks such as classification, identification, and change detection of spectral data [29,30]. SAM performs sample matching and identification by comparing the spectral angles between a target sample and known samples. Although SAM is one of the most classic and traditional algorithms, it is also more reliable and offers higher flexibility and operability in model updates. Furthermore, it is insensitive to changes in brightness and lighting. Therefore, in this study, SAM was utilized to match the targets in the directly smeared urine samples with known samples in the database to determine the presence of bacterial targets in the directly smeared samples. The intuitive results of SAM make the results of positive–negative determination models easier to understand and interpret. The specific calculation formula for SAM is as follows:

(5) $$\alpha =\frac{\sum_{i=1}^{n_{b}}t_{i}r_{i}}{{\left(\sum_{i=1}^{n_{b}}{t_{i}}^{2}\right)}^{\frac{1}{2}}{\left(\sum_{i=1}^{n_{b}}{r_{i}}^{2}\right)}^{\frac{1}{2}}}$$

Among them, *n_b_* is the number of spectral bands, *t* and *r* represent the reference spectrum and the test spectrum, respectively. The spectral cosine is used as a similarity measure, which measures the similarity between the reference spectrum and the test spectral vector based on the angle between them. A smaller angle indicates a higher similarity in spectral features, while a larger angle indicates greater dissimilarity.

### 2.6. MBNet

In the field of medical imaging, most end-to-end models based on three-dimensional (3D) convolutional networks are proposed for processing stereoscopic imaging modes such as Computed Tomography (CT) and Magnetic Resonance Imaging (MRI) [31]. These imaging modes have the characteristics of relatively simple semantic features and fixed organ structures, which are somewhat different from the high variability and complexity of micro-hyperspectral images. In micro-hyperspectral analysis, 3D convolution means performing convolution operations by sliding the convolution kernel in three directions: two spatial directions and one spectral direction [32,33]. The definition of 3D convolution is shown in Equation (6):

(6) $$F(i,j,h)=\left(K*I\right)(i,j,h)=\sum_{m,n,p}I(i,j,h)*K(i-m,j-n,h-p)$$

In this equation, F(i,j,h) represents the output 3D feature map, I(i,j,h) is the input 3D vector, and K(i,j,h) is the 3D convolution kernel. i,j,  and h represent the coordinates of the three output directions, namely the positions of spatial dimension, spatial dimension row, and spectral dimension column. m,n,and p represent the sizes of the convolutional kernel in these three directions, and these three parameters collectively determine the receptive field size of that layer. The use of 3D convolution is more suitable for extracting features from datacubes, as it not only extracts spatial features but also spectral features.

**Figure 4:**
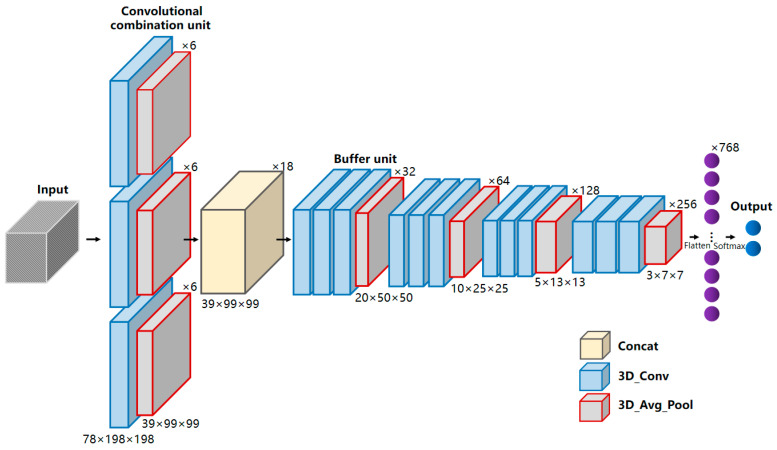
Model architecture of MBNet. MBNet mainly includes a convolutional combination unit, a feature concatenation layer, and four buffer units. The convolutional combination unit consists of three convolutional kernels with different dimensions, and each buffer unit consists of three convolutional layers and one downsampling layer. Among them, the yellow square represents the concatenation layer, the blue squares represent the 3D convolutional layers, and the gray squares represent the 3D average pooling layers.

Therefore, constructing a determination model based on 3D convolution is not a simple linear combination of 1D and 2D convolutional networks. Due to the inherent difficulty in obtaining medical samples, overfitting is prone to occur when directly applying deeper models. The deeper network models have more parameters, higher complexity, and are more challenging to train, which contradicts the limited sample size of the directly smeared urine sample in this study. A CNN network with relatively fewer layers and parameters may be more suitable for this study. Therefore, this study proposed a multi-scale convolutional neural network, the Multi-BufferNet (abbreviated as MBNet). Its model architecture is shown in Figure 4.

The term “multi-scale” refers to using convolutional kernels of different sizes to process the same layer of feature maps, combining different convolutional kernels in a parallel manner, and merging the convolutional results. The use of different convolutional kernels is to introduce receptive fields of different sizes and extract features at various scales. To address the characteristics of rich spectral information and varying target sizes in directly smeared urine data, a convolutional combination unit consisting of three sets of convolutional kernels was designed. The convolutional combination unit stacks 3 × 1 × 1, 3 × 3 × 3, and 3 × 5 × 5 (spectral dimension λ × spatial dimension row × spatial dimension column) kernels together. The 3 × 1 × 1 kernel is dedicated to extracting spectral information, while the 3 × 3 × 3 and 3 × 5 × 5 kernels are used to capture spatial texture information at different scales. The feature maps obtained from the three sets of convolutional kernels are concatenated in the feature concatenation layer to produce the output feature maps. Subsequently, a combined buffer unit of the buffer layer and the downsampling layer was designed, with a downsampling stride of 2. The stride of the buffer layer is fixed at 1 to enhance representational power without reducing the feature map’s resolution. Convolutional kernels are all 3 × 3 × 3 in size to extract detailed information of datacubes. After the feature concatenation layer, four buffer units are sequentially connected, and finally, a fully connected layer and a SoftMax layer are passed to output the determination results.

### 2.7. Evaluation Metrics

This study employed Accuracy (ACC), Positive Predictive Value (PPV), and negative predictive value (NPV) as evaluation metrics. ACC is often used as a measure of classification performance in hyperspectral image analysis, while PPV and NPV are often applied in the medical field. PPV represents the proportion of true positive results among those that were determined as positive during diagnosis or testing. NPV represents the proportion of true negative results among those that were determined as negative. The specific calculation formulas for these metrics are as follows:

(7) $$ACC=\frac{TP+TN}{TP+TN+FP+FN}$$

(8) $$PPV=\frac{TP}{TP+FP}$$

(9) $$NPV=\frac{TN}{TN+FN}$$

**Figure 5:**
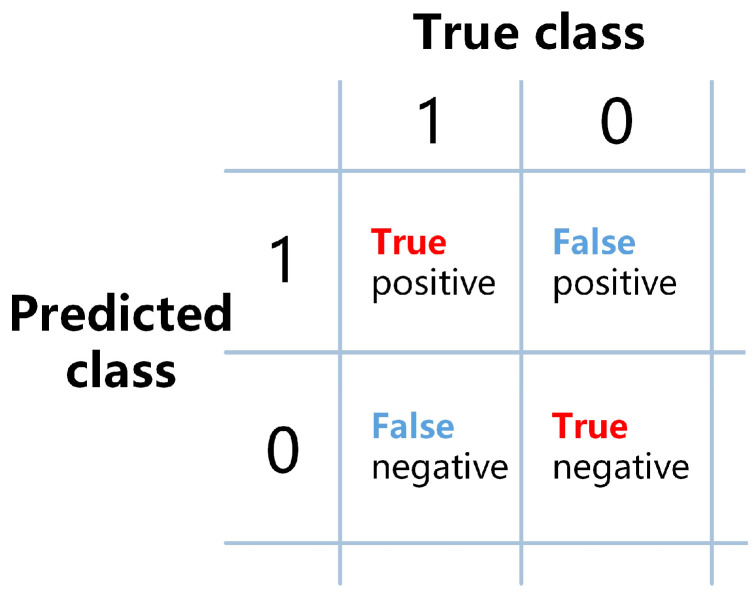
Schematic diagram of the relationship between *TP*, *FP*, *TN*, and *FN*.

*TP*, *FP*, *TN*, and *FN* represent the number of samples that are true positives (correctly predicted positive), false positives (incorrectly predicted positive), true negatives (correctly predicted negative), and false negatives (incorrectly predicted negative), respectively. The relationships between these metrics are shown in Figure 5.

## 3. Results and Discussion

### 3.1. Hyperspectral Database Matching of Bacterial Sample

#### 3.1.1. Hyperspectral Database of Directly Smeared Urine Sample

**Figure 6:**
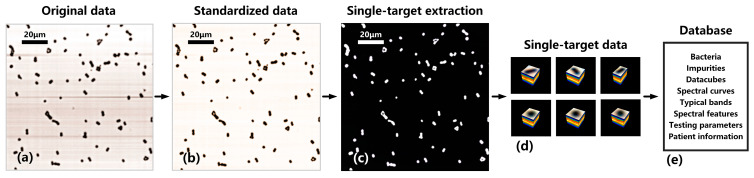
Database standardization process for directly smeared hyperspectral data. (**a**) Original data of a directly smeared bacterial sample. (**b**) Standardized data after standardized correction. (**c**) Binary image of target and background. (**d**) Single-target datacubes after single-target extraction. (**e**) The data content contained in the database.

The database standardization process for directly smeared hyperspectral data is shown in Figure 6. Figure 6a represents the original image of directly smeared bacterial samples, and Figure 6b represents the image after standardized correction. From Figure 6a, it can be seen that due to the influence of factors such as system light source, optical components, and experimental environment, there are a large number of horizontal shadows in the original image. These shadows not only introduce errors during image processing but also obscure bacterial features, resulting in the loss of some important bacterial features. After standardized correction, as shown in Figure 6b, the situation with horizontal shadows is significantly improved, and each bacterial feature is clearly visible.

The goal of this study is to determine whether the directly smeared urine sample is infected with bacteria, that is, whether there are bacteria present in the sample. Therefore, in order to quickly identify bacteria, we need to obtain a hyperspectral datacube of a single bacterium (single target). On the basis of standardized data, we used the K-means clustering algorithm to separate the foreground target and background region in which the number of clusters, initialization, and iterations were set to 2, 10, and 200, respectively. Then, a binary image of the target and background was obtained, as shown in Figure 6c. Combining bacterial morphological parameters, we removed impurities and unknown bacteria with abnormal parameters in the foreground. Additionally, we excluded targets (incomplete bacteria and impurities) at the image boundary and retained the remaining single targets as data samples. Then, we identified connected regions in the binary image and accurately located and selected single-target samples from the datacube based on their positions in the binary image. By setting several constraints such as single-target size range, aspect ratio range, and restrictions on a single connected region, the automatic extraction of single-target datacubes can be achieved, as shown in Figure 6d. The obtained single-target samples have the same dimension of spectral band but different spatial dimensions due to variations in their own spatial sizes.

Finally, a hyperspectral database of directly smeared bacteria was established, as shown in Figure 6e. On the one hand, the database includes standard strains, genus, and species information of clinical bacterial samples, microscopic datacubes, standard spectral curves, typical spectral bands, visual spectral features, medical testing parameters, and basic patient information. On the other hand, to address the interference from directly smeared impurities (and others), a hyperspectral database of directly smeared impurities, including urine cells, casts, and crystals, was established.

#### 3.1.2. SAM Results of Directly Smeared Bacteria

**Figure 7:**
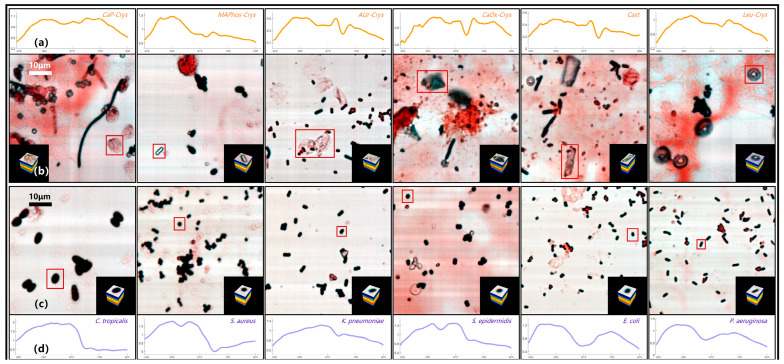
Pseudo-color images and typical spectral curves of directly smeared samples. (**a**) Typical spectral curves of different impurities. (**b**) Pseudo-color images and datacubes of different impurities. (**c**) Pseudo-color images and datacubes of different bacteria. (**d**) Typical spectral curves of different bacteria.

Figure 7 shows the pseudo-color images and typical spectral curves of common targets in the directly smeared sample database, including several typical bacterial data and several representative impurity data. The impurities in Figure 7 contain flaky calcium phosphate crystal (*CaP-Crys* for short), magnesium ammonium phosphate crystal (*MAPhos-Crys*), ammonium urate crystal (*AUr-Crys*), calcium oxalate crystal (*CaOx-Crys*), *Cast*, and leucine crystal (*Leu-Crys*). The bacteria in Figure 7 contain *C. tropicalis*, *S. aureus*, *K. pneumoniae*, *S. epidermidis*, *E. coli*, and *P. aeruginosa*. Among them, the large images in Figure 7b,c are pseudo-color images containing the targets. The red boxes represent the locations of the targets, and the small images in the lower right or left corners are the datacubes extracted from the corresponding targets. It can be seen that positive samples with higher bacterial content usually have relatively fewer impurities, while negative samples tend to have a higher impurity content.

**Figure 8:**
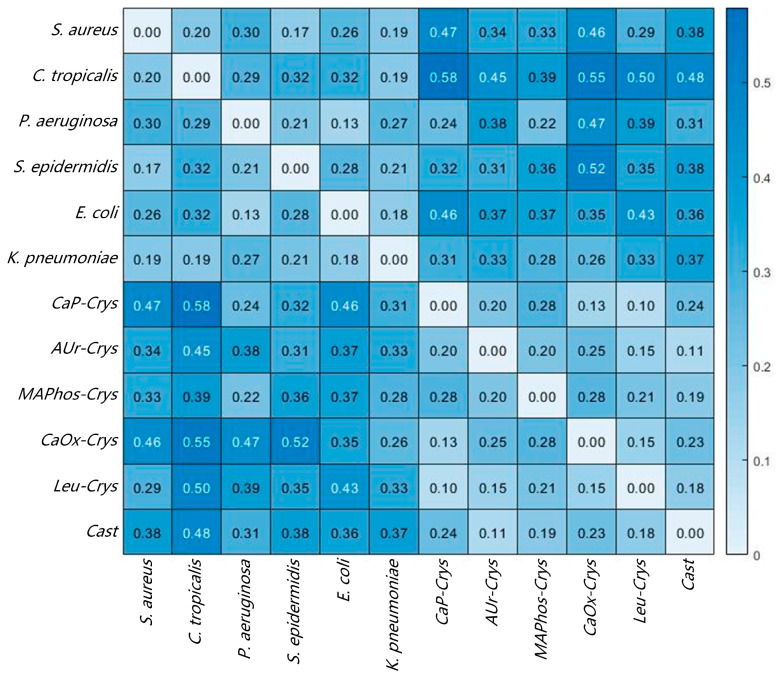
SAM results of different bacteria and impurities.

The images in Figure 7a,d represent the typical spectral curves of the corresponding target, with the horizontal axis representing the wavelength. Overall, there are significant differences in the spectral curve features between bacteria and impurities. However, there are some similar spectral features between several bacteria or impurities. For example, *S. aureus* and *S. epidermidis* both have a clear feature valley at the 60 spectral band (590 nm), which may be related to the fact that they both belong to the Staphylococcus genus [27]. *E. coli* and *P. aeruginosa* show similar trends in their spectral curves. *AUr-Crys* and *Cast* also share a continuous feature peak and valley between 90 and 110 spectral bands (660–705 nm). In order to have a clearer understanding of the spectral feature similarities between several common bacteria and impurities in Figure 7, SAM analysis was conducted for the above targets. The spectral curve of a specific bacterium or impurity was used as the reference spectrum, and the spectral curves of the other bacteria and impurities were treated as the test spectra. The typical spectral curve is derived by averaging the spectral curves of various bacteria in the database. The spectral angles between these spectra were calculated, and the results are shown in Figure 8. The closer the spectral angle is to 0, the more similar the spectral features are. The diagonal has values of 0 because the reference spectrum and the test spectrum are the same spectral curve.

Overall, the spectral angle values mostly fall within the range of 0.2 to 0.5. Values smaller than 0.2 indicate relatively similar spectral features, which mostly occur between bacteria of the same genus or among impurities, such as between *S. aureus* and *S. epidermidis*, as well as *Leu-Crys* and *CaP-Crys*. Values greater than 0.5 indicate significant differences, mostly occurring between bacteria and impurities. Further analysis reveals that the spectral angles between similar targets and typical spectral curves in the database were mostly less than 0.1. Therefore, in order to use SAM to determine similarity and better balance accuracy and coincidence rate, the determination threshold was set to 0.1; that is, if the spectral angle between the test target and a known substance is lower than this threshold, the matching result for this target will be output. If the spectral angle between the test target and a known substance is higher than this threshold, it is considered as no matching result.

### 3.2. Determination of Positive–Negative Bacterial Infection Based on MBNet

**Figure 9:**
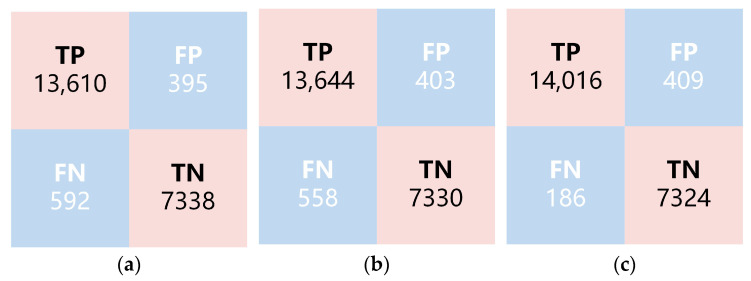
Results of different determination models. (**a**) MBNet-0 h; (**b**) MBNet-3 h; (**c**) Joint Model.

MBNet takes hyperspectral data of directly smeared urine samples (160 × 400 × 400) as the model input. First, the convolution combination unit is set with strides of (2, 2, 2), and the 6 resulting feature maps are pooled and concatenated into 18 feature maps (39 × 99 × 9). The spectral dimension is downsampled to compress the spectral information. The output consists of 64 feature maps with a size of 98 × 9 × 9. Then, it proceeds through four buffer combination units in sequence, and the output feature maps with sizes of 20 × 50 × 50, 10 × 25 × 25, 5 × 13 × 13, and 3 × 7 × 7. The number of output feature maps is 32, 64, 128, and 256, respectively. During model training, 40% of the total samples are used as the training set, with 30% being the test set and 30% being the validation set. The training samples are shuffled in each iteration. The training batch is set to 64, the learning rate is set to 0.001, and the stochastic gradient descent is used as the optimizer. After passing through the four buffer combination units, the model outputs 256 feature maps with a size of 3 × 7 × 7. Finally, a fully connected layer is used to output two probability values for bacterial infection status, either positive or negative. After 80 iterations of the training, the model parameters were determined. The validation set data were input into the model for validation, and the results are shown in Figure 9a.

The validation set consists of 21,935 samples, with results of 13,610 TP, 7338 TN, 395 FP, and 592 FN. The performance of the model was evaluated by calculating accuracy, PPV, and NPV, as shown in Table 2. MBNet achieved an average accuracy of 95.50%, indicating that MBNet’s multi-scale buffer architecture is suitable for the analysis and determination of 3D datacubes. The four-layer buffered structure enhances the feature representation capability with limited downsampling. The combination of three parallel convolutional kernels, while balancing model complexity and accuracy, extracts spectral features of directly smeared data from different dimensions, playing a positive role in extracting multi-scale positive–negative features of bacterial samples. PPV and NPV reached 97.18% and 92.54%, respectively. Due to a relatively higher proportion of negative samples being misclassified as positive, the NPV value is relatively lower. Considering the model’s advancement, uniqueness, and versatility, VGGNet [34], ResNet [35], DenseNet [36], and Vision Transformers (ViT) [37] models were selected for training to further demonstrate the performance of MBNet. For all comparison methods, the training and testing data were set to be the same. The results are shown in Table 2. MBNet achieved superior results while its model was lighter, which is more conducive to practical application.

To test the impact of short-term cultivation on the positive–negative determination and further validate the model performance, the 0 h samples used in the above experiments were replaced with 3 h samples for model training and validation. The 3 h and 0 h samples remained consistent in terms of data collection, preprocessing, model architecture, and training mode. The difference is that the 3 h samples were prepared from the original samples after a 3 h short-term culture, rather than being directly smeared. The results of MBNet-3 h are shown in Figure 9b and Table 2. Compared with the results of MBNet-0 h, MBNet-3 h showed a slight improvement in both ACC and NPV, while PPV decreased slightly. This may be related to the increase in bacterial counts after short-term cultivation, which corrected some of the previously misclassified positive samples. However, overall, the improvement in each metric was not significant. Considering the time and labor costs associated with the 3 h cultivation, the feasibility of selecting 0 h samples is higher in practical applications. On the other hand, whether 0 h or 3 h samples are used, their NPV values are relatively low. NPV provides the probability of correctly identifying true negatives under negative results and is a metric for evaluating diagnostic or testing accuracy. A higher negative coincidence rate means that there is a higher level of confidence in correctly identifying true negatives under a negative diagnosis, which is relatively more important in clinical practice.

### 3.3. Joint Determination of Positive–Negative Bacterial Infection

**Figure 10:**
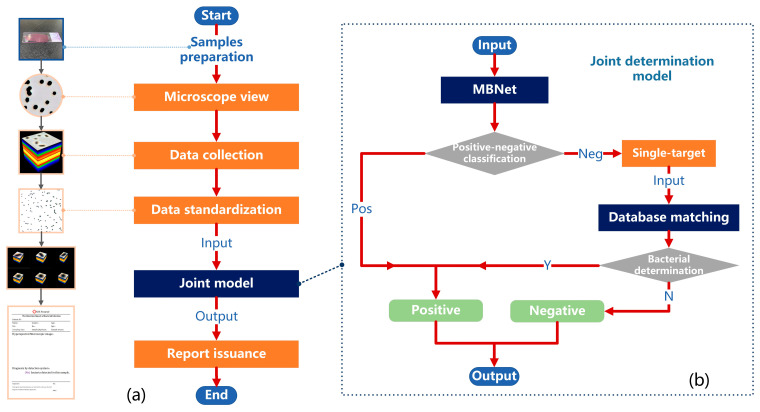
The determination solution of the whole process for positive–negative bacterial infection. (**a**) The flow chart of determination solution. (**b**) The flow chart of the joint determination model.

In order to further enhance the NPV of positive–negative determination and improve clinical applicability, this section combines MBNet-based determination with bacterial spectral database matching to propose a more efficient model for the joint determination of positive–negative bacterial infection. The determination process of the joint model is shown in Figure 10b, mainly including MBNet classification, single-target automatic extraction, and spectral database matching. Firstly, the standardized bacterial hyperspectral data are divided into four 160 × 400 × 400-sized sample data, which are input into MBNet for positive–negative classification. For samples classified as positive, the results are directly output, while for those classified as negative, single-target automatic extraction is performed. In the connected regions of the binary image, impurities with abnormal parameters, unknown targets, and targets at the image boundary (incomplete bacteria or impurities) are excluded, while retaining the datacubes of other single targets. Then, the average spectral curve of the single target is extracted, and it is matched with the standard spectral curves of each substance in the database. That is, if the spectral angle between the test target A and a substance B in the database is lower than the set threshold, the target A will be determined as B. If B belongs to a known bacterial species, it is determined to be bacterial. If B belongs to impurities or other substances, it is determined to be non-bacterial. If the spectral angle between test target A and all known substances in the database is above the threshold, it is determined as a no-match result, meaning non-bacterial. Once a bacterial determination (Y for Yes in Figure 10b) occurs during the single-target matching process, the output is classified as positive. If no bacterial determination (N for No in Figure 10b) occurs, the output is classified as negative.

The validation results for the joint model are shown in Figure 9c and Table 2. Compared with MBNet-0 h, the joint model demonstrated a significant improvement in accuracy, reaching 97.29%, and NPV improved by 5.06% to reach 97.60%. This substantial improvement is attributed to the effective combination of MBNet with spectral database matching. For MBNet, its model architecture focuses more on global or large-sized features, which may overlook scattered single bacterial targets in some regions. Spectral database matching focuses more on the characteristics of small targets. The combination of single-target extraction and spectral angle matching is beneficial in improving the sensitivity of the original model to small targets. After spectral angle matching, single bacterial targets that were previously overlooked were discovered, resulting in the correction of a large number of positive samples that were misclassified as negative. The number of FN samples decreased from 592 to 186, leading to a significant improvement in NPV.

As a result, we were able to establish a rapid determination solution of the whole process for positive–negative bacterial infection. As shown in Figure 10a, the entire solution starts with the preparation of directly smeared (urine) samples and ends with the issuance of a test report (including basic database information and test results). The specific steps are as follows:

1. Prepare the directly smeared samples as described in Section 2.2.
2. Observe the entire field of view under the microscope and locate the appropriate area.
3. Collect the hyperspectral data of urine samples potentially infected with bacterial/fungal via MICROspecim.
4. Standardize hyperspectral data as described in Section 2.4.
5. Input data into the joint model to determine the bacterial infection (positive or negative).
6. If the result is negative, issue a detection report stating “No bacteria detected in this sample.” If the result is positive, issue a detection report stating “Bacteria detected in this sample.”

The entire process, except for the sample centrifugation, takes approximately 5–10 min. The software operation can be completed within 3–5 min, where the data collection process (Step 2) takes 2 min, and the data analysis process (Step 3, Step 4, and Step 5) takes 1–3 min. During the joint determination, if MBNet is determined to be positive, the result will be directly generated. If it is negative, further database matching is required, which relatively takes more time. Except for Step 1, all other software operations are completed in the micro-hyperspectral acquisition system. The overall acquisition system is primarily developed in C#. In addition to the main functions of data collection, functions such as data standardization preprocessing, database integration, joint model determination, and result visualization are all uniformly implemented through Python. This part packages the relevant models and the trained weight files developed in Python as dynamic link library (.exe) files. Subsequent upgrades and updates can be quickly achieved by replacing the .exe files. This determination solution has been put into clinical practice, and we have found that laboratory doctors attach more importance to the metric of NPV. If NPV can be further improved on the existing basis, it will allow clinical laboratories to exclude samples determined as negative by the system, focusing only on samples determined as positive, which can greatly shorten the time required to issue the final report.

## 4. Conclusions

This study addressed the rapid determination of positive–negative infection in directly smeared bacterial samples by proposing a novel solution that combines micro-hyperspectral imaging with deep learning models. First, a standard hyperspectral database of common bacteria and impurities in urine samples was established. To extract the spectral features of directly smeared data from different dimensions, MBNet with a multi-scale buffered network was proposed. MBNet achieved an average accuracy of 95.50%, indicating that the combination of multiple parallel convolutional kernels and buffered architecture is suitable for the analysis of bacterial directly smeared data. In order to improve the sensitivity of MBNet to small targets in bacterial samples, a joint determination model was developed by combining a spectral database matching algorithm. This joint model achieved an accuracy of 97.29%, a PPV of 97.17%, and a NPV of 97.60%. Finally, this study established a rapid determination solution by combining the directly smeared samples preparation and software analysis reports, which is also an exploration of more efficient and feasible bacterial rapid detection technologies. In addition, through the continuous accumulation of directly smeared data at present, we plan to extend this technology to the rapid testing of sterile body fluids, such as pleural effusion and ascites. Furthermore, for positive samples, we plan to conduct targeted model training on their two-dimensional spatial features to determine the content of bacteria on the sample carrier, thereby inferring their infection degree, which is also the focus of our future research.

## Figures and Tables

**Table 1:** The types and numbers of samples in the experimental dataset.

Infection Status		Urine Sample	Experimental Sample
**Negative**		2864	25,776
**Positive**	*E. coli*	1442	12,978
	*K. pneumoniae*	510	4590
	*A. baumannii*	236	2124
	*P. mirabilis*	365	3285
	*E. faecalis*	720	6480
	*S. epidermidis*	322	2898
	*P. aeruginosa*	315	2835
	*S. aureus*	296	2664
	*C. albicans*	460	4140
	*C. tropicalis*	594	5346
**Total**		8124	73,116

**Table 2:** Comparison of metrics for the validation results of different determination models.

Model	ACC/%	PPV/%	NPV/%
MBNet-0 h	95.50	97.18	92.54
VGGNet	91.54	93.00	88.77
ResNet	91.30	92.61	88.79
DenseNet	92.71	94.12	90.08
ViT	94.74	96.16	92.18
MBNet-3 h	95.62	97.13	92.93
Joint Model	97.29	97.17	97.60

## Data Availability

Data will be made available by the corresponding author upon reasonable request (https://zenodo.org/doi/10.5281/zenodo.10473650).

## References

[1] Yang P., Wang X. (2020). COVID-19: A new challenge for human beings. Cell. Mol. Immunol..

[2] Baker R.E., Mahmud A.S., Miller I.F., Rajeev M., Rasambainarivo F., Rice B.L., Takahashi S., Tatem A.J., Wagner C.E., Wang L.-F. (2022). Infectious disease in an era of global change. Nat. Rev. Microbiol..

[3] Bloom D.E., Cadarette D. (2019). Infectious disease threats in the twenty-first century: Strengthening the global response. Front. Immunol..

[4] Chen H., Liu K., Li Z., Wang P. (2019). Point of care testing for infectious diseases. Clin. Chim. Acta..

[5] Wang H., Ceylan Koydemir H., Qiu Y., Bai B., Zhang Y., Jin Y., Tok S., Yilmaz E.C., Gumustekin E., Rivenson Y. (2020). Early detection and classification of live bacteria using time-lapse coherent imaging and deep learning. Light Sci. Appl..

[6] Ombelet S., Barbé B., Affolabi D., Ronat J.-B., Lompo P., Lunguya O., Jacobs J., Hardy L. (2019). Best practices of blood cultures in low-and middle-income countries. Front. Med..

[7] Li H., Hsieh K., Wong P.K., Mach K.E., Liao J.C., Wang T.-H. (2023). Single-cell pathogen diagnostics for combating antibiotic resistance. Nat. Rev. Methods Primers.

[8] Garner C., Brazelton de Cardenas J., Suganda S., Hayden R. (2021). Accuracy of broad-panel PCR-based bacterial identification for blood cultures in a pediatric oncology population. Microbiol. Spectr..

[9] Behzadi P., Urbán E., Matuz M., Benkő R., Gajdács M. (2021). The role of gram-negative bacteria in urinary tract infections: Current concepts and therapeutic options. Adv. Microbiol. Infect. Dis. Public Health..

[10] Tullus K., Shaikh N. (2020). Urinary tract infections in children. Lancet.

[11] Paoletti M., Haut J., Plaza J., Plaza A. (2019). Deep learning classifiers for hyperspectral imaging: A review. ISPRS J. Photogramm. Remote Sens..

[12] Stuart M.B., McGonigle A.J., Willmott J.R. (2019). Hyperspectral imaging in environmental monitoring: A review of recent developments and technological advances in compact field deployable systems. Sensors.

[13] Lv W., Wang X. (2020). Overview of hyperspectral image classification. J. Sens..

[14] Feng L., Zhu S., Liu F., He Y., Bao Y., Zhang C. (2019). Hyperspectral imaging for seed quality and safety inspection: A review. Plant Methods.

[15] Özdoğan G., Lin X., Sun D.-W. (2021). Rapid and noninvasive sensory analyses of food products by hyperspectral imaging: Recent application developments. Trends Food Sci. Technol..

[16] Du J., Tao C., Xue S., Zhang Z. (2023). Joint Diagnostic Method of Tumor Tissue Based on Hyperspectral Spectral-Spatial Transfer Features. Diagnostics.

[17] Yoon J. (2022). Hyperspectral imaging for clinical applications. BioChip J..

[18] Zheng L., Wen Y., Ren W., Duan H., Lin J., Irudayaraj J. (2022). Hyperspectral dark-field microscopy for pathogen detection based on spectral angle mapping. Sens. Actuators B Chem..

[19] Soni A., Dixit Y., Reis M.M., Brightwell G. (2022). Hyperspectral imaging and machine learning in food microbiology: Developments and challenges in detection of bacterial, fungal, and viral contaminants. Compr. Rev. Food Sci. Food Saf..

[20] Eady M., Setia G., Park B. (2019). Detection of Salmonella from chicken rinsate with visible/near-infrared hyperspectral microscope imaging compared against RT-PCR. Talanta.

[21] Liu K., Ke Z., Chen P., Zhu S., Yin H., Li Z., Chen Z. (2021). Classification of two species of Gram-positive bacteria through hyperspectral microscopy coupled with machine learning. Biomed. Opt. Express..

[22] Kang R., Park B., Eady M., Ouyang Q., Chen K. (2020). Single-cell classification of foodborne pathogens using hyperspectral microscope imaging coupled with deep learning frameworks. Sens. Actuators B Chem..

[23] Kang R., Park B., Ouyang Q., Ren N. (2021). Rapid identification of foodborne bacteria with hyperspectral microscopic imaging and artificial intelligence classification algorithms. Food Control.

[24] Park B., Seo Y., Yoon S.-C., Hinton A., Windham W.R., Lawrence K.C. (2015). Hyperspectral microscope imaging methods to classify gram-positive and gram-negative foodborne pathogenic bacteria. Trans. ASABE.

[25] Michael M., Phebus R.K., Amamcharla J. (2019). Hyperspectral imaging of common foodborne pathogens for rapid identification and differentiation. Food Sci. Nutr..

[26] Ho C.-S., Jean N., Hogan C.A., Blackmon L., Jeffrey S.S., Holodniy M., Banaei N., Saleh A.A., Ermon S., Dionne J. (2019). Rapid identification of pathogenic bacteria using Raman spectroscopy and deep learning. Nat. Commun..

[27] Tao C., Du J., Tang Y., Wang J., Dong K., Yang M., Hu B., Zhang Z. (2022). A Deep-Learning Based System for Rapid Genus Identification of Pathogens under Hyperspectral Microscopic Images. Cells.

[28] Li Y.-H., Tan X., Zhang W., Jiao Q.-B., Xu Y.-X., Li H., Zou Y.-B., Yang L., Fang Y.-P. (2021). Research and application of several key techniques in hyperspectral image preprocessing. Front. Plant Sci..

[29] Peng J., Sun W., Ma L., Du Q. (2019). Discriminative transfer joint matching for domain adaptation in hyperspectral image classification. IEEE Geosci. Remote Sens. Lett..

[30] Chang C.-I. (2021). Hyperspectral target detection: Hypothesis testing, signal-to-noise ratio, and spectral angle theories. IEEE Trans. Geosci. Remote Sens..

[31] Villarraga-Gómez H., Herazo E.L., Smith S.T. (2019). X-ray computed tomography: From medical imaging to dimensional metrology. Precis. Eng..

[32] Li Q., Wang Q., Li X. (2021). Exploring the relationship between 2D/3D convolution for hyperspectral image super-resolution. IEEE Trans. Geosci. Remote Sens..

[33] Li W., Chen H., Liu Q., Liu H., Wang Y., Gui G. (2022). Attention mechanism and depthwise separable convolution aided 3DCNN for hyperspectral remote sensing image classification. Remote Sen..

[34] Simonyan K., Zisserman A. (2014). Very deep convolutional networks for large-scale image recognition. arXiv.

[35] He K., Zhang X., Ren S., Sun J. Deep residual learning for image recognition. Proceedings of the IEEE Conference on Computer Vision and Pattern Recognition.

[36] Huang G., Liu Z., Van Der Maaten L., Weinberger K.Q. Densely connected convolutional networks. Proceedings of the IEEE Conference on Computer Vision and Pattern Recognition.

[37] Dosovitskiy A., Beyer L., Kolesnikov A., Weissenborn D., Zhai X., Unterthiner T., Dehghani M., Minderer M., Heigold G., Gelly S. (2020). An image is worth 16x16 words: Transformers for image recognition at scale. arXiv.

